# Descriptive and molecular analysis of pineal parenchymal tumors with clinical correlation

**DOI:** 10.1186/s12885-025-15331-1

**Published:** 2025-12-06

**Authors:** Hend Khaled, Amal Mosaab, Hala Taha, Amal Refaat, Nada Ashraf, Mohamed Saad Zaghloul, Mohamed El-Beltagy, Olfat Ahmad, Manal Zamzam, Mark W. Kieran, Ahmed El-Hemaly, Shahenda El-Naggar, Alaa El-Haddad

**Affiliations:** 1 Pediatric Oncology Department, Children’s Cancer Hospital, Egypt 57357, 1 Sekket El Emam, El Madbah El Kadeem Yard, Cairo, Sayeda Zeinab Egypt; 2 Tumor Biology Research Program, Basic Research Unit, Research Department, Children’s Cancer Hospital, Egypt 57357, 1 Sekket El Emam, El Madbah El Kadeem Yard, Sayeda Zeinab, Cairo, Egypt; 3 Department of Pathology, Children’s Cancer Hospital, Egypt 57357, Cairo, Egypt; 4 Department of Pathology, National Cancer Institute, Cairo University, Cairo, Egypt; 5 Department of Radiodiagnosis, Children’s Cancer Hospital, Egypt 57357, Cairo, Egypt; 6 Department of Radiodiagnosis, National Cancer Institute, Cairo University, Cairo, Egypt; 7 Department of Clinical Research, Children’s Cancer Hospital, Egypt 57357, Cairo, Egypt; 8 Department of Radiation Oncology, Children’s Cancer Hospital, Egypt 57357, Cairo, Egypt; 9 Department of Radiation Oncology, National Cancer Institute, Cairo University, Cairo, Egypt; 10 Department of Neurosurgery, Children’s Cancer Hospital, Egypt 57357, Cairo, Egypt; 11 Department of Neurosurgery, Kasr Al-Ainy School of Medicine, Cairo University, Cairo, Egypt; 12 King Hussein Cancer Center (KHCC), Amman, Jordan; 13 Division of Pediatric Neuro-oncology, Hopp Children’s Cancer Center (KiTZ), Heidelberg, Germany; 14 Department of Pediatric Oncology, National Cancer Institute, Cairo University, Cairo, Egypt

**Keywords:** Pineoblastoma, DNA methylation, Pineal parenchymal body tumors, PPTID KBTBD4-altered, BRD4-LEUTX fusion

## Abstract

**Background:**

Pineal parenchymal body tumors are rare central nervous system tumors with a variety of presentations ranging from well-differentiated low-grade tumors to undifferentiated highly aggressive tumors. Recent molecular classification has described this heterogeneity, particularly among pineoblastomas (PB) and pineal parenchymal tumor of intermediate differentiation (PPTID).

**Methods:**

Our study analyzed 49 patients with pineal parenchymal tumors, including PBs (*n* = 39), papillary tumors of the pineal region (*n* = 5), PPTID (*n* = 2), pineocytoma (*n* = 1), and trilateral retinoblastoma (*n* = 2). Descriptive analysis of patients’ characteristics was done in percentages and numbers. Overall survival (OS) and event-free survival (EFS) analysis were evaluated in relation to age and metastatic status for PB cases. Molecular classification was performed using the EPIC methylation array and analyzed by Heidelberg Classifier on 20 cases, of which sixteen were histopathologically diagnosed as PB.

**Results:**

Among PBs, univariate analysis showed that age significantly impacted OS and EFS (p-value = 0.003 and 0.021, respectively), while metastatic status only impacted EFS (p-value = 0.032). In Multivariate analysis, only age was of significance on OS (p-value 0.028). The identified methylation groups were PB-miRNA-1 (*n* = 10), PB-RB1 (*n* = 1), retinoblastoma-MYCN activated (*n* = 1), PPTID KBTBD4-altered (*n* = 1), papillary tumor of the pineal region (*n* = 1), medulloblastoma (MB) WNT activated (*n* = 1), MB non-WNT/SHH (*n* = 1), CNS embryonal tumor with BRD4-LEUTX fusion (*n* = 1) and unclassified N/A (*n* = 3).

**Conclusion:**

Our data identified age as a prognostic factor affecting survival among our PB cohort. We also highlighted the heterogeneity of pineal parenchymal body tumors, necessitating molecular classification for accurate diagnosis and for developing tailored treatment strategies. We demonstrate the feasibility of identifying new entities and MBs within pineal body tumors, thereby supporting the growing evidence that MBs originate in the pineal region.

**Clinical trial number:**

Not applicable.

**Supplementary Information:**

The online version contains supplementary material available at 10.1186/s12885-025-15331-1.

## Introduction

Pineal gland tumors are rare and aggressive neoplasms, accounting for less than 3% of all primary brain tumors in children and adolescents [1]. According to the World Health Organization (WHO) classification 2021, pineal tumors are classified into several subtypes: pineocytoma, papillary tumor of the pineal region (PTPR), pineal parenchymal tumor of intermediate differentiation (PPTID), pineoblastoma (PB), and desmoplastic myxoid tumor (DMT) of the pineal region SMARCB1-mutant [2]. Pineocytomas are slow-growing WHO grade 1 neoplasms; they are well-circumscribed and locally confined [3]. Total surgical resection is the main line of treatment without adjuvant therapy [4]. PTPRs are rare WHO grade 2 & 3 neuroepithelial tumors [5]. The optimal treatment for PTPR remains controversial. Cases are primarily treated with total resection; adjuvant radiotherapy (RTH) or chemotherapy is mostly spared for recurrent or metastatic cases [4, 6].

PPTID is a group of pineal tumors WHO grade 2 & 3, characterized by being intermediate between pineocytomas and PBs [3]. Recently WHO 2021 has included in-frame small insertions in the KBTBD4 gene (Kelch repeat and BTB domain-containing protein 4) as a desirable criterion for diagnosis of PPTID [2] Histologically, grade 2 PPTID retains a high neurofilament expression, similar to pineocytomas, but with a Ki67/MIB-1 index of 6% to 10%, higher than pineocytomas [3, 7] While PPTID grade 3 retains a minimal neurofilament expression and an Ki67/MIB-1 index ranges from 10 to 20% [3]. Treatment of PPTID remains controversial due to the rarity of the tumor and the lack of a uniformly treated patient population. Adjuvant RTH is recommended in all patients who underwent partial or subtotal resection, and craniospinal irradiation (CSI) is generally recommended for grade 3 tumors [3].

PBs are WHO grade 4 tumors, representing 40% of parenchymal pineal body tumors [5]. They mostly occur sporadically, but a few can occur in the context of cancer predisposition syndromes, the main ones being DICER1 and RB1 germline variants [4]. In case of DICER1, PB harbor complete inactivation of DICER1 activity in contrast to other DICER1 syndrome-related tumors, which arise without total loss of DICER1 function [4]. This biallelic inactivation occurs by a combination of a loss-of-function mutation coupled with a loss of heterozygosity (LOH)/chromosome 14q loss [8]. While in the case of RB germline variant, there is about 5% risk of developing trilateral retinoblastoma, i.e., pineoblastoma associated with concomitant bilateral (or very rarely unilateral) retinoblastoma [4, 8]. PBs are aggressive, poorly differentiated, rapidly growing tumors, with a tendency for leptomeningeal dissemination in 15% of patients [3]. Histologically, they contain Homer-Wright rosettes with central neuropil [7]. They also express high Ki67/Mib1 index from 20 to 25% to 50% [3, 7]. Their main line of treatment is surgical resection followed by CSI and chemotherapy [3]. The DMT SMARCB1-mutant tumors of the pineal region are a new entity with distinct clinical, histopathological, and molecular features showing epigenetic similarities with ATRT-MYC [9].

Recent advancements in methylation profiling have revealed significant heterogeneity within pineal parenchymal tumors. They are categorized into different molecular consensus groups: PB-miRNA1, PB-miRNA2, PB-MYC/FOXR2, PB-RB1, and PPTID. Age at diagnosis and metastatic status vary significantly among these groups. Patients with PB-miRNA2 have superior outcomes with a 5-year overall survival (OS) of 100% in comparison to PB-miRNA1 (5-year OS of 67.5%), PB-MYC/FOXR2 (5-year OS of 20.5%), and PB-RB (5-year OS of 26.8%) [10].

This report describes clinical and molecular classification of cases from Children’s Cancer Hospital in Egypt (CCHE) with pineal parenchymal tumors based on DNA methylation signatures. Our findings support that these tumors are diverse, with distinct clinical and molecular features and variable outcomes.

## Materials and methods

### Tumor sample selection and clinical data

All patients diagnosed with pineal parenchymal tumors at the CCHE between 2007 and 2022 were included in this study. The patient population consisted of 49 patients for whom complete clinical and response data were available. Data was obtained after approval from the CCHE Institutional Review Board for waiver of consent due to the study's retrospective nature and the use of anonymized patient data. After central review, tumor specimens were collected from the Department of Pathology as formalin-fixed paraffin-embedded tissue (FFPE). The diagnosis is based on the WHO 2021 classification criteria. Histological diagnoses of pineal parenchymal tumors were made by assessing variables such as small round blue cells with hyperchromatic nuclei, Homer-Wright or Flexner-Wintersteiner rosettes, mitosis, karyorrhexis, nuclear-cytoplasmic ratio (N/C), synaptophysin positivity, and Ki-67 labeling index.

### Genome-wide DNA methylation profiling

Genomic DNA extraction from the FFPE samples was performed with a detailed description in a prior publication [11]. In brief, DNA isolation was done using QIAamp DNA FFPE tissue kit (Qiagen) and quantified using DENOVIX Fluorometer (dsDNA High Sensitivity). The quality of the DNA was assessed using the Illumina FFPE QC kit (Illumina Inc.). A minimum of 250 ng of extracted DNA was used to proceed with bisulfite conversion. EZ DNA methylation kit (D5002, Zymo Research) was used for bisulfite conversion of the extracted DNA. The bisulfite-converted DNA was restored using the Infinium HD FFPE DNA Restore Kit (WG-321–1002, Illumina Inc.). Restored bisulfite-converted DNA was then hybridized to the Illumina Infinium Human Methylation EPIC 850 K bead chips and scanned using the Illumina iScan microarray scanner according to the manufacturer’s recommendations (Illumina Inc.).

### Methylation analysis

DNA methylation analysis was performed using the Illumina Infinium MethylationEPIC BeadChip arrays. Methylation-based tumor classification was conducted using the latest version of the Heidelberg classifier (v12.8), based on the published [12] random forest classification algorithm. Tumor samples were projected onto a suitable reference cohort from the Molecular Neuro-Pathology (MNP) reference set from the German Cancer Research Center (DKFZ). The t-distributed stochastic neighbor embedding (t-SNE) plot was initially generated using the DKFZ’s in-house Shiny application, and further edited using RStudio (v4.2.0) and Inkscape. (Methylation data are available through Gene Expression Omnibus (GEO: http://www.ncbi.nlm.nih.gov/geo/), accession number GSE269319).

### PCR based detection of KBTBD4 insertion

The case showing KBTBD4 insertion by methylation was further analyzed by PCR. KBTBD4 insertion detected by conventional PCR using the following primers (forward: AAACAGTTTGTGCCACCAGA, reverse: ATATGGCATCTTTCCCGGG). The PCR products were size-separated by electrophoresis on a 4% agarose gel in 1X Tris-Borate-EDTA (TBE) buffer for 1 h. A 100 bp DNA ladder was used for band size estimation.

### Staging of patients

All patients had an initial brain and whole spine magnetic resonance imaging (MRI), followed by maximum safe resection. The extent of resection was defined as gross total resection (GTR) if there was no detectable tumor on postoperative images; near-total resection (NTR) if more than 95% of the tumor was resected with evidence of residual tumor; subtotal resection (STR) if tumor resection was between 50% and 95%; partial resection if resection was between 10% and 50% of the tumor; and biopsy if resection was less than 10% of the tumor. Lumbar cerebrospinal fluid (CSF) analysis was done on day 14 post-surgery [13].

### Treatment protocols

Treatment of PB patients was according to age. Patients < 3 years received maximum safe resection followed by chemotherapy according to the COG P9934 protocol [14]. The induction phase of chemotherapy (cisplatin, vincristine, cyclophosphamide, and etoposide) was followed by focal conformal radiotherapy of 54 Gy. CSI was preserved only for metastatic patients after a multidisciplinary team discussion, followed by four cycles of maintenance chemotherapy (vincristine, cyclophosphamide, and etoposide). All PB patients ≥ 3 years old were treated according to the COG ACNS0332 protocol [15]. They were subjected to maximum safe resection followed by CSI of 36 Gy with tumor bed boost to reach 55.8 Gy, followed by six cycles of maintenance chemotherapy (vincristine, cyclophosphamide, and cisplatin). Patients diagnosed with trilateral retinoblastoma received treatment according to ARET0321 protocol [16]; patients received four cycles of chemotherapy (vincristine, cyclophosphamide, cisplatin, and etoposide) followed by marrow-ablative high - dose chemotherapy and autologous hematopoietic cell rescue (HDCT/AuHCR) and radiotherapy. Other parenchymal pineal body tumors were treated according to the multidisciplinary team’s decision.

## Response evaluation

Response to treatment evaluation was done at the end of induction and end of treatment by MRI of the whole brain and spine. Response criteria were categorized as follows: Complete response (CR) was defined as the complete disappearance of all assessable tumors, partial response (PR) is defined as a greater than 50% decrease in the tumor size, stable disease (SD) is defined as a decrease in the tumor size between 25% and 50% or less than a 25% increase in the tumor size, and progressive disease (PD) is defined as the appearance of any new tumor lesions or greater than a 25% increase in tumor size [13].

### Statistical analysis

Descriptive analysis of patients’ characteristics was reported in numbers and percentages. Fisher’s exact test was used to assess the distribution of metastatic status among different age groups in PBs. Survival analysis was calculated using Cox Regression analysis. Overall survival was defined as the period from the date of registration to the date of death from any cause or last follow-up​. In contrast, event-free survival (EFS) was defined as the period from the date of registration to the occurrence of the first event, which included disease progression, recurrence, or death from any cause, or until the date of the last follow-up for patients who did not have events. A log-rank test was employed to compare the outcomes; a p-value < 0.05 was considered significant. Statistical analysis was done using SPSS version 25.

## Results

### Clinical and molecular characteristics of the study cohort

This retrospective study included clinical and treatment responses of 49 patients diagnosed with pineal parenchymal tumors. Clinical and histopathological features of the cohort are described in Table 1. The male-to-female ratio was 1.6:1. The range of age at diagnosis was 1 to 15.9 years. PB represented the majority of the cohort, 79.5% (*n* = 39). Only three patients had STR while the remaining patients had a stereotactic biopsy. Patients ≥ 3 years represented 76.9% (*n* = 30), while patients < 3 years represented 23.1% (*n* = 9). Among the patients with papillary tumor of the pineal region, PPTID, and pineocytoma, all presented with localized disease regardless of their tumor grade. In contrast, the two trilateral retinoblastoma (TRB) patients presented with spinal seedlings along with the development a pineal lesion.

**Table 1:** Clinical description of the study cohort

Patients’ Characteristics	Number (N= 49)	Percent (%)
Gender		
Male	30	61%
Female	19	39%
Age at Diagnosis		
Mean	7.4	
Median	6.6	
<3	9	19%
≥3	40	81%
Pathology		
PB, WHO grade 4	39	80%
PTPR, WHO grade 2	3	6%
PTPR, WHO grade 3	2	4%
Trilateral RB	2	4%
PPTID, WHO grade 2	1	2%
PPTID, WHO grade 3	1	2%
Pineocytoma	1	2%
Metastatic Status		
M+	18	37%
M0	31	63%
Extent of resection		
Biopsy	46	93.8%
STR	3	6.12%

*Abbreviations:* *PB* Pineoblastoma, *PTPR* Papillary Tumor of the Pineal Region, *RB* Retinoblastoma, *PPTID* Pineal Parenchymal Tumors of Intermediate Differentiation, *STR* Subtotal Resection

**Fig. 1:**
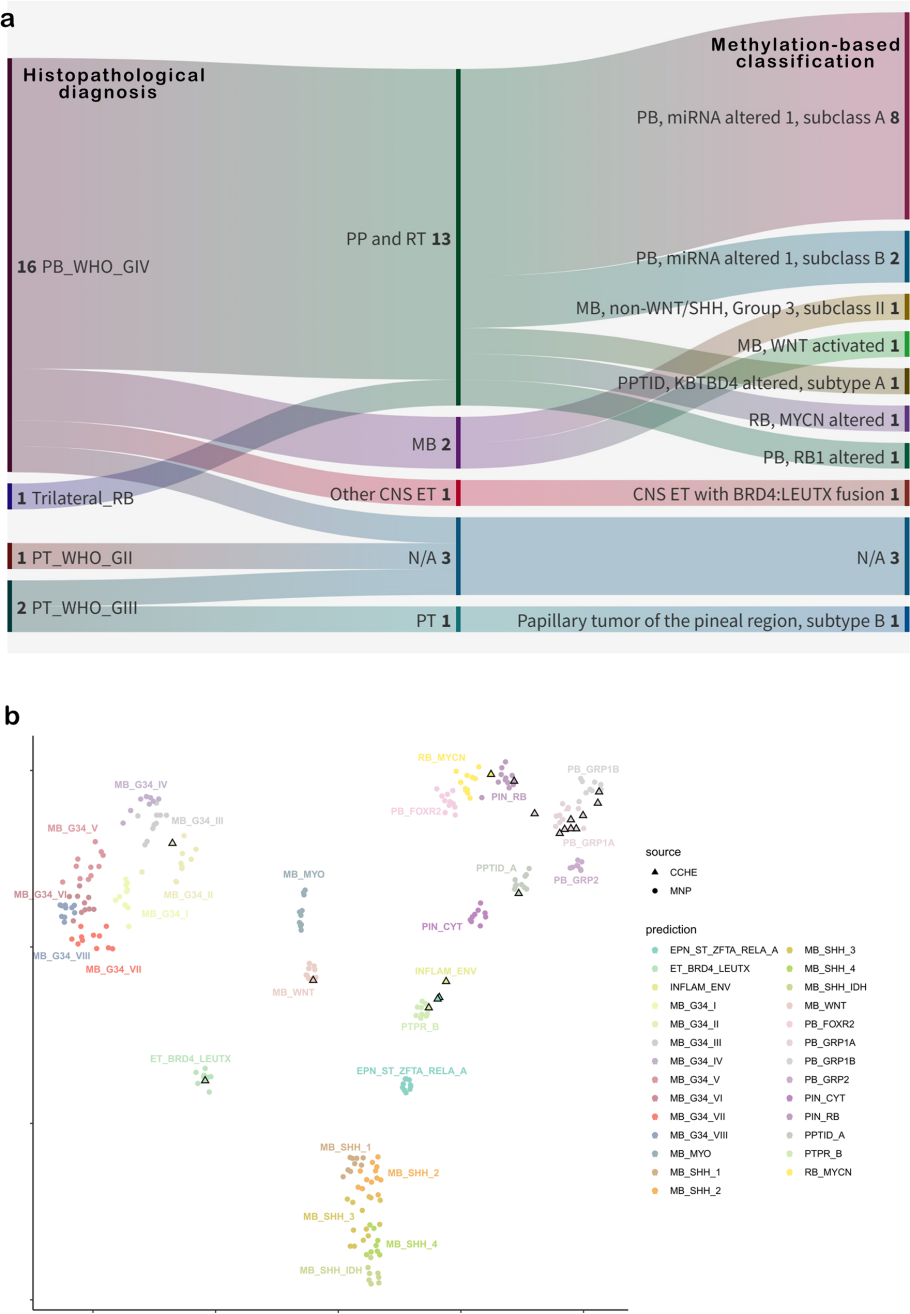
Molecular Classification of Pineal Body Tumors. **a** Sanky plot for histopathological diagnosis (left) for 20 pineal parenchymal tumors and the corresponding superfamily (middle) and subclass (left) identified by DNA methylation-based classification by the Heidelberg classifier version 12.8. PT, pineal tumor; PB, Pineoblastoma; RB, Retinoblastoma; MB, Medulloblastoma; PP, Pineal Parenchymal; CNS, Central Nervous System; ET, Embryonal Tumor; PPTID, Pineal Parenchymal Tumor of I ntermediate Differentiation. **b** A t-distributed stochastic neighbor embedding (t-SNE) plot showing DNA methylation profiles of the 20 pineal parenchymal tumors from CCHE against a suitable background cohort from the DKFZ Molecular Neuro-Pathology (MNP) reference set

Among the 49 patients, only 20 had sufficient DNA for methylation array testing. The 20 cases, distributed initially among four histopathological classes, were reallocated into nine distinct molecular subgroups (Table 2) (Fig. 1a). t-SNE was then used to visualize DNA methylation classes for the 20 tumor samples against a suitable background from the MNP reference set of DKFZ (Fig. 1b). All tumor samples clustered with their corresponding classes. The calibrated classification scores for subclasses (Supplementary Table 2) were > 0.9 for all samples except for the MB- non WNT non SHH Group 3 subclass II, PPTID KBTBD4-altered, and one of the PB miRNA-1 subclass A (pin_02) (scoring 0.63, 0.83, and 0.69, respectively). The MB- non-WNT non-SHH scored 0.99 for the superfamily and clustered with MBs on the t-SNE. Similarly, PPTID KBTBD4-altered subtype A scored 0.93 for the superfamily and clustered with its corresponding class. Although pin_02 scored the least among PB miRNA-1, it still clustered with the same group. It was also observed that PB-RB1 was characterized by a loss of ch16 (Supplementary Fig. 1a) and RB-MYCN displayed ch6p gain (Supplementary Fig. 1b).

**Table 2:** Clinical and molecular correlation of the methylated samples

Patients’Characteristics	DNA-based methylation subclasses								
	PB-miRNA1(n=10)	PB-RB1(n=1)	RB -MYCN(n=1)	PTPR(n=1)	PPTID KBTBD4-altered(n=1)	MB WNT(n=1)	MB non WNT- non SHH(n=1)	BRD4-LEUTX(n=1)	N/A(n=3)
Age									
< 3 years	-	-	1	-	-	-	-	1	-
≥ 3 years	10	1	-	1	1	1	1	-	3
Gender									
Males	7	-	1	-	-	1	1	1	3
Females	3	1	-	1	1	-	-	-	-
Metastatic status									
M+	6	1	1	-	-	-	-	1	-
M0	4	-	-	1	1	1	1	-	3
Histopathology									
PB	10	-	1	-	1	1	1	1	1
PTPR Grade 2	-	-	-	-	-	-	-	-	1
PTPR Grade 3	-	-	-	1	-	-	-	-	1
Trilateral Rb	-	1	-	-	-	-	-	-	-

*Abbreviations:* *PB* Pineoblastoma, *PTPR* Papillary Tumor of the Pineal Region, *RB* Retinoblastoma, *PPTID* Pineal Parenchymal Tumors of Intermediate Differentiation, *MB* Medulloblastoma and *N/A* Not classified

### Association of clinical and molecular variables with patients’ outcomes

Among the thirty PB patients aged ≥ 3 years, 66.6% (*n* = 20) had non-metastatic disease, and 33.3% (*n* = 10) had metastatic disease. All patients started treatment as per protocol, except one who died before initiating treatment due to postoperative complications. Among the < 3 Years age group, 77.7% (*n* = 7) of the patients were metastatic, and 22.3% (*n* = 2) were non-metastatic, of which only two were alive at the last follow-up. One patient lacked enough data regarding the cause of death and died before initiating treatment. Additional clinical data are provided in Supplementary Table 1. Univariate Cox proportional hazards analysis demonstrated that age significantly impacted both OS (*p*-value = 0.003) and EFS (*p*-value = 0.021), whereas metastatic status showed a significant association only with EFS (*p*-value = 0.032) (Table 3). In the multivariate analysis including age and metastatic status, age remained an independent predictor of OS (*p*-value = 0.028) while metastatic status lost statistical significance (Table 3). Assessment of the extent of resection was not performed since most of the patients were biopsied.

**Table 3:** Univariate and multivariate cox regression analysis

Variables	OS				EFS			
	No. of events/No. of cases	HR	95% CI	***P*** -value	No. of events/No. of cases	HR	95% CI	***P*** -value
**Univariate Cox Regression Analysis**								
Age Group	39				39			
≥ 3 years	30/39	1	-	-	30/39	1	-	-
< 3 years	9/39	4.606	1.671–12.690	0.003*	9/39	3.219	1.193–8.683	0.021*
Metastatic Status	39				39			
Non-metastatic	23/39	1	-	-	23/39	1	-	-
metastatic	16/39	2.221	0.867–5.694	0.097	16/39	2.712	1.090–6.748	0.032*
**Multivariate Cox Regression Analysis**								
Age Group	39				39			
≥ 3 years	30/39	1	1.166–15.854	0.028*	30/39	1		
< 3 years	9/39	4.3			9/39	2.054	0.617–6.842	0.241
Metastatic Status	39				39			
Non-metastatic	23/39	1			23/39	1		
Metastatic	16/39	1.106	0.325–3.761	0.871	16/39	1.956	0.645–5.933	0.236

Estimated hazard ratio for overall and event-free survival with 95 % confidence interval and p value of the likelihood ratio test, (*) is added for p-value less than 0.05

*Abbreviations:* *OS* Overall Survival, *EFS* Event free Survival, *HR* Hazard Ratio, *CI* Confidence Interval

Regarding the patients with papillary tumor of the pineal region (*n* = 5), one patient (grade 2) received upfront focal RTH (54 Gy) and remained stable without progression at the last follow-up (8 years). In contrast, the remaining four cases progressed locally, received salvage focal RTH (54 Gy), and remained alive at their last clinic visits (follow-up period: 8.1 months, 4, 4.3, and 6.5 years). For the two patients with PPTID, the patient diagnosed as grade 3 received upfront focal RTH (54 Gy), then developed local and distant progression and died of disease after 7 months. Conversely, the patient diagnosed as grade 2 received no treatment and has remained with stable disease for 8 years. While the single patient with pineocytoma was kept under follow-up, they remained stable and alive for 8 years since diagnosis. Both cases of trilateral retinoblastoma had poor outcomes; both died during induction chemotherapy due to progressive disease, and neither received HDCT/AuHCR or RTH.

Patients classified as miRNA-1 (*n* = 10) were all histologically classified as PBs and within the same age group (range 5.2–15.9 years). The patient with PB-RB1-altered died 2 months after developing the pineal tumor. Also, the patient with RB-MYCN-altered had a poor outcome. Their time to progression varied (28.55 and 11.64 months, respectively), and both died of progressive disease. The case diagnosed as PPTID KBTBD4-altered was histologically diagnosed as PB. Further validation by conventional PCR to detect the insertion elicited a larger band with 197 bp as compared to the wild type 188 bp (Supplementary Fig. 2), indicating the insertion. Further analysis by sequencing could not be performed due to insufficient DNA. The patient maintained a progression-free survival for 68.8 months. Lastly, the patient diagnosed with a papillary tumor of the pineal region maintained a progression-free survival for 9.7 months till the last assessment.

### Identification of ectopic Medulloblastoma in the pineal region

**Fig. 2:**
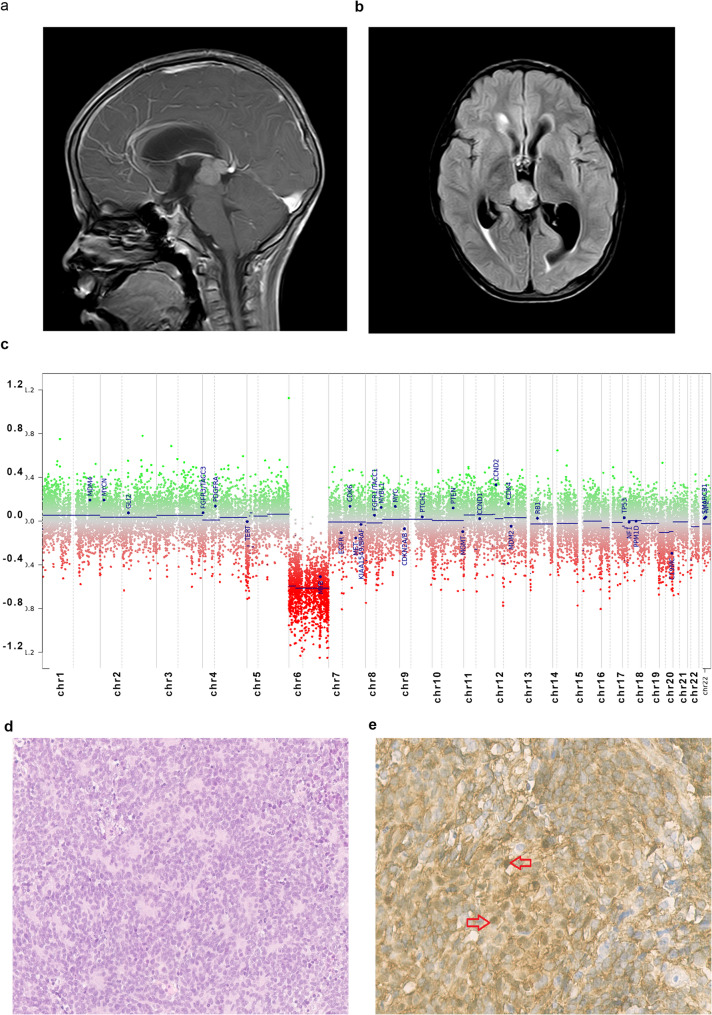
Medulloblastoma WNT-activated radiological, CNV profile and histopathological features. **a** Sagittal T1-weighted image post contrast and **(b)** FLAIR sequence of MRI brain, both demonstrating a well-defined mass with moderate enhancement centered upon the pineal gland measuring 2.3 × 2.1 × 2.1 cm. **c** Copy number variation (CNV) profile. DNA methylation profiling analyses by the Heidelberg Classifier V12.8 show loss of chromosome 6 (https://www.molecularneuropathology.org). **d** H&E staining. **e** Beta-catenin immunohistochemistry respectively (40x). Nuclear accumulation of beta-catenin in a proportion of tumor cells (red arrows)

Two cases were molecularly classified as MB. The first case was a male patient ≥ 3 years of age who presented with a localized pineal body mass proven radiologically by MRI (Fig. 2a and b). A biopsy was performed, and histologically, it was compatible with PB. Treatment was administered according to the COG ACNS0332 protocol. The patient was in CR at the end of the treatment and remained disease-free for 51 months at his last clinic visit. DNA-methylation-based analysis classified the sample as MB-WNT (prediction score 0.99). Copy number variation analysis demonstrated loss of chromosome 6 (monosomy 6) (Fig. 2c). This was further confirmed by nuclear ß-catenin accumulation by IHC, supporting WNT-pathway molecular diagnosis activation (Fig. 2d).

**Fig. 3:**
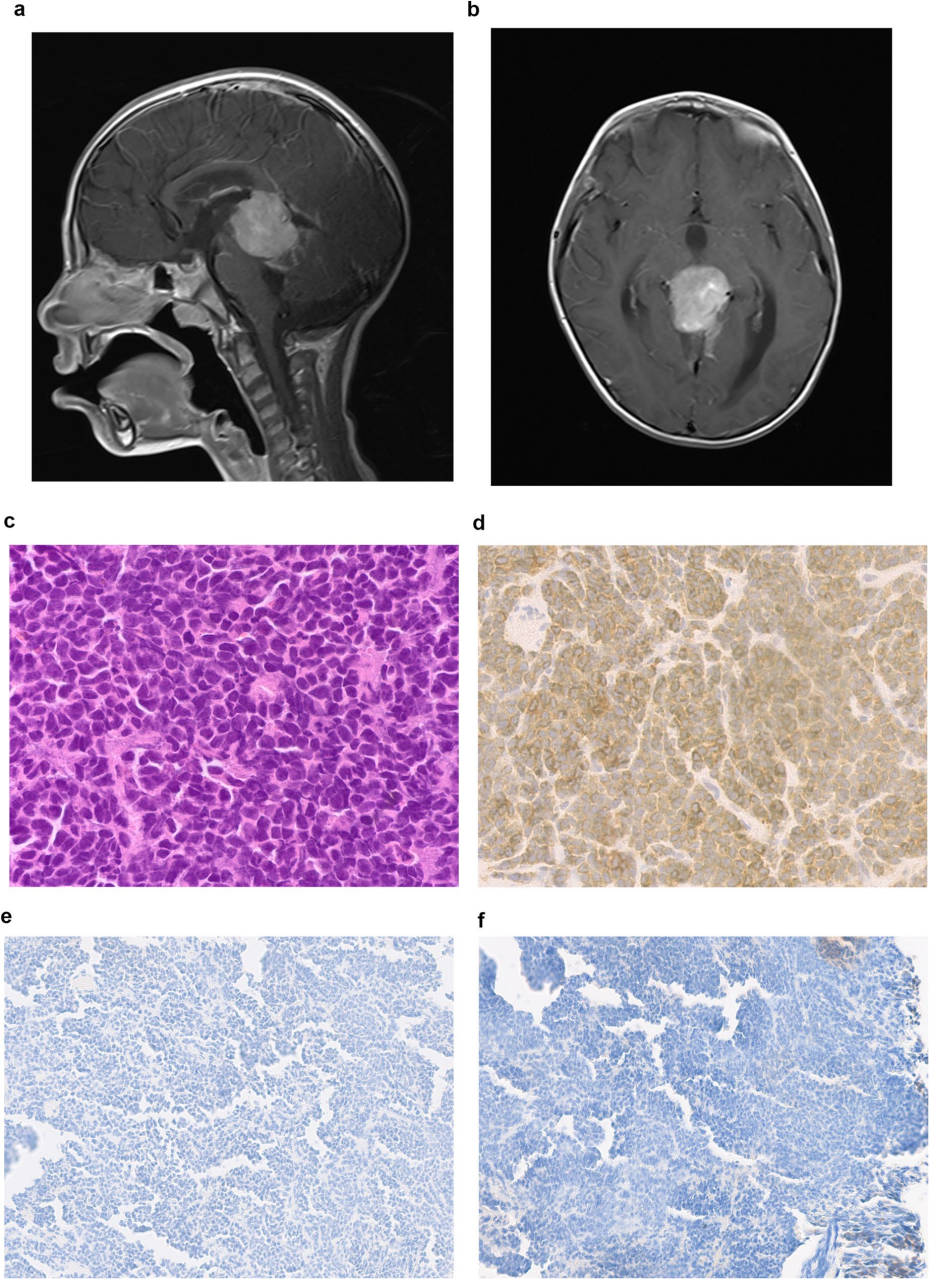
Medulloblastoma non WNT-non SHH, radiological and histopathological features. **a** Sagittal T1-weighted post contrast and **(b)** axial T1-weighted images of MRI brain post contrast, both demonstrating a well-defined lesion in the pineal region with intense enhancement measuring 3.3 × 3 × 3.5 cm. **c** H&E staining with mitosis karyorrhexis and few rosettes (40x). **d** Diffuse positive cytoplasmic reaction to synaptophysin (40x). **e**,** f **Shows no immunoreactivity to YAP1 and GAB1 stains respectively (20x)

The second case was a male, ≥ 3 years of age, who presented with a localized pineal mass. The patient had a STR, and the tumor was histologically diagnosed as PB. The patient received treatment according to the COG ACNS0332 protocol. The patient was in CR at the end of treatment but developed a distant relapse 8 months later and died of disease progression. DNA-methylation analysis classified the sample as MB non-WNT/SHH, Group 3, Subclass II IHC was then performed for YAP1 and GAB1. Both stains were negative, supporting the diagnosis of MB non-WNT/SHH (Fig. 3e and f).

### Identification of CNS embryonal tumor with ՙՙBRD4-LEUTX fusion՚՚

**Fig. 4:**
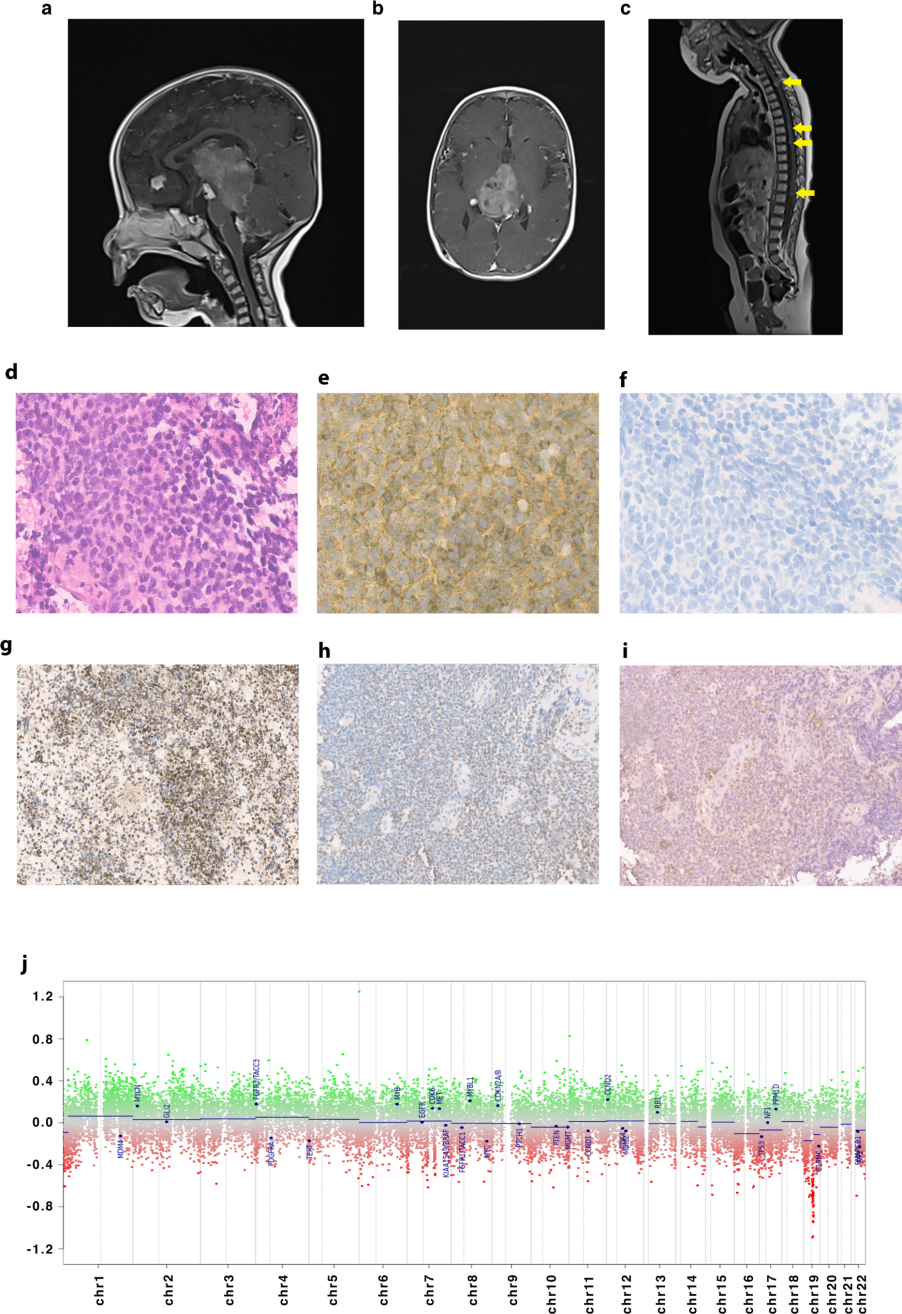
CNS embryonal tumor with BRD4: LEUTX fusion, radiological histopathological features and CNV profile. **a** Sagittal T1-weighted post contrast and **(b)** axial T1 MRI brain post contrast, demonstrating a pineal body mass measuring 4 × 3.4 × 4.8 cm and multiple areas of nodular leptomeningeal seedlings.​ **c** MRI ​spine post contrast, sagittal T1- weighted showing leptomeningeal dissemination (yellow arrows). **d** H&E staining (40x). **e** Synaptophysin immunohistochemical positive reaction(40x). **f** PLAP immunohistochemical negativity (40x). **g** Retained INI (20x). **h** Focally retained ATRX expression (20x). **i** Focally retained nuclear expression of SMARCA4 (20x). **j** CNV profile of DNA methylation profiling analyses by the Heidelberg Classifier V12.8 show no specific chromosome gains or losses. (https://www.molecularneuropathology.org)

The patient was a male < 3 Years of age with a large pineal mass (measuring 4 × 3.4 × 4.8 cm), with multiple supra, infratentorial, and spinal seedlings (Fig. 4a b, and 4c). The tumor was histologically diagnosed as PB. By microscopy, the cells were small to medium-sized and exhibited a high N/C ratio with brisk mitotic activity, karyorrhexis, and scattered rosettes. By IHC, the tumor demonstrated diffuse positivity to synaptophysin, PLAP negativity, retained INI-1, focally retained ATRX expression, and focally retained nuclear expression of SMARCA4 (Fig. 4e and i). Furthermore, the H3K27m mutation was negative for mutation. While RNA sequencing could not be performed due to the poor quality of available tissue. DNA-methylation-based analysis classified the sample as a CNS embryonal tumor with BRD4-LEUTX fusion “novel entity” (prediction score = 0.99). The CNV was uniform, with no significant gains or losses (Fig. 4j). From a clinical perspective, the patient received only 2 cycles of induction chemotherapy according to the COG P9934 protocol and died of disease progression 4 months after diagnosis.

## Discussion

Pineal parenchymal tumors are uncommon neuroepithelial neoplasms with diverse features, grades, and, more recently, molecular entities [10]. Due to their rarity, only a few studies have described the management of this group of patients. Herein, we retrospectively analyzed the clinical and pathological characteristics of our cohort and utilized a methylation array for molecular classification.

In our cohort, most cases were PB (79.5%). Only the impact of age and metastatic status on patients’ outcomes was analyzed, while the degree of resection could not be assessed, as the majority of patients were biopsied. By univariate analysis, age group (< 3 years and ≥ 3 years) significantly impacted both OS and EFS, while the metastatic status significantly impacted EFS. However, in multivariate analysis, only age maintained significance in OS. The lack of significance of metastases in the multivariate analysis could be attributed to the limited number of patients; hence, a larger patient cohort would be required for proper assessment. The poor outcome of patients < 3 years may be explained by their increased likelihood of presenting with metastatic disease and high progression rate as compared to the elder group. This reflects the more aggressive biology behind these tumors as observed in earlier studies [13, 17].

SJYC07 [18] and Head Start (I-III) [13] trials offered different treatment options to patients < 3 Years, including radiotherapy sparing and HDCT, to spare young patients the neurocognitive deficits related to radiotherapy, but with unsatisfactory outcomes. On the other hand, Mynarek et al. [17] reported a few patients < 4 years with localized disease who survived with HDCT and focal radiotherapy. Conclusions on the optimum treatment for this group can’t yet be made. This group of patients remains challenging, requiring a balance between limiting toxicities and controlling an aggressive disease behavior.

Our study showed better outcomes with patients ≥ 3 years, especially with localized disease with RTH and standard chemotherapy dose. Series such as SJMB03 [19] achieved a 5-year OS/PFS of 100% by adopting tandem HDCT/AuHCR and a lower CSI dose (23.4 Gy) to nonmetastatic cases. While data pooled from SIOP and Head start [14] did not show the same impact of HDCT with elder patients, HDCT was only of benefit in metastatic disease. While comparison between studies is difficult, the question of whether high-dose chemotherapy maybe a treatment option for this group remains controversy. This diversity of clinical presentations and outcomes among PBs warrants consideration of future molecularly-driven treatment protocols for this rare pediatric brain tumor entity [9, 19].

Methylation analysis was only performed on 20 of the 49 patients; this limitation is attributed to the scarcity of the tissue samples. Among the 20 samples, 16 were histologically diagnosed as PB. The 16 cases were subclassified into seven different methylation classes. miRNA-1 represented 62.5% (*n* = 10/16), while miRNA-2 was not detected in our cases. All ten cases were in the same age group (≥ 3 years). Notably, 6 of the 10 cases had metastatic disease at presentation. Our results are consistent with previous reports describing miRNA-1 and miRNA-2 molecular groups in the group ≥ 3 years [10]. Moreover, patients with miRNA-1 had a higher metastatic potential. Regarding outcome, miRNA subtypes of PB are thought to have a good prognosis, especially in localized disease [10]. However, due to small numbers in other molecular classes in our cohort, such comparison between different classes was not attainable.

Methylation analysis classified one of the tumor samples as RB-MYCN activated subtype (prediction score = 0.95), an entity that was not previously described in the pineal body. This was a case of 18 months old patient, histologically diagnosed as PB, presented with metastatic disease, subsequently progressed and died within a year from diagnosis. Our patient had no evidence of a retinal tumor. This entity is described by the classifier as being different from typical RB as it lacks the inactivation of RB1, but shares a CNV of 6p gain a feature of retinoblastoma. It shows amplification of MYCN, overlapping the PB-MYC/FOXR2 subtype. Clustered in proximity to both PB-RB and PB-FOXR2. This case overlaps clinically with both PB-RB and PB-FOXR2 by being aggressive and young in age. Molecularly it shares features of both. We hypothesize that this tumor arises from a primitive photoreceptive progenitor cell common to both the pineal gland and retina [4, 20] hence carrying both features with *MYCN* amplification driving its aggressive behavior. Regardless of the nomenclature, this class points to an aggressive disease and requires further studies to identify its nature and the suitable line of treatment.

The molecularly defined PPTID KBTBD4-altered patient was histologically diagnosed as PB. Diagnosing PPTID presents a diagnostic challenge as it carries histological and biological features mimicking well-differentiated pineocytoma and poorly differentiated PB. Recent molecular classification reported in-frame insertion in the KBTBD4 gene as a characteristic finding of PPTIDs [2, 9]. We further analysed our case by PCR. Results obtained showed a broader band as compared to wild type indicating the insertion. Proceeding with sequencing couldn’t be achieved due to insufficient remaining DNA. This technique can be of benefit in case there is insufficient DNA for molecular analysis or if methylation is unavailable. A recent study by Rahmanzade R et al. [21] suggested that PPTIDs can be classified into two sub-groups: PPTIDs with KBTBD4 insertion harboring a small-cell morphology with an unfavorable clinical course and PPTIDs without KBTBD4 insertions with a large-cell morphology and a favorable clinical course. In addition, Ki67 ≥ 8% also suggested a worse outcome. Such data was based on a limited sample size and will require expanded analysis of more patients. Our patient harbored the KBTBD4 alteration, had a Ki67 >8%, was treated as PB with CSI and chemotherapy, and remained in remission. Although the patient carried unfavorable features as described by Rahmanzade R et al. [21] He still had a favorable outcome in line with the consensus report [9]. As this was a single case, it is not possible to determine whether disease control was due to treatment efficacy or favourable biological background. Looking into our other two cases histologically diagnosed as PPTID, we will find the first case diagnosed as grade 2 remained under follow without treatment and survived without disease progression. On contrary, the other case with grade 3 progressed locally and distantly. Unfortunately, a uniform comparison couldn’t be made between the 3 cases as they weren’t all molecularly defined. This highlights the importance of molecular analysis in such a case to unify the diagnosis and study how these tumors respond to treatment.

Two of our cases had methylation patterns consistent with MB, even though both were located in the pineal region. Our first case was classified as MB WNT-activated, and the second classified as non-WNT/non-SHH MB. The molecular diagnosis of MB in regions other than the posterior fossa has recently been reported, both in the pineal body [19, 22] and in the sellar region [23]. This data argues that MB is no longer exclusive to the posterior fossa. Liu et al. reported 6 MB-WNT cases in the pineal body; three patients were grossly resected, and four received reduced RTH doses [22]. Despite the different management modalities, all cases survived without evidence of recurrence. This is in line with our patient, who was biopsied, received CSI (36 Gy), and stayed in remission. Meanwhile, recent trials (ACNS1422, PNET5, and SJMB12) [24] are now evaluating chemotherapy and CSI dose reduction for MB-WNT with favorable features. Accordingly, methylation analysis should be applied as part of the pineal body tumor diagnostic panel to reduce RTH/chemotherapy for such a group of patients.

A single case of PB carrying the BRD4: LEUTX fusion was identified. This entity is described by the Heidelberg classifier and has not yet been integrated into the WHO CNS classification yet. Nine cases harboring this fusion within a CNS tumor were recently reported in a case series [25]. The clinical presentation of our case goes in line with that described in the series, in being of young age and in the presence of disseminated disease. In terms of clinical follow up, our case shortly died with disease progression which was similar to some of the cases yet there were cases which responded to chemotherapy with complete resolution of the disease. By histology, our case shared similar high grade features and by IHC our case shared positivity of synaptophysin, retained ATRX and retained SMARCA4. By methylation our case shared a flat copy number profile. Bromodomain-containing protein 4 (BRD4), a member of the bromodomain and extra terminal (BET) protein family, is important in controlling oncogene expression and genome stability. Abnormal expression and dysfunction of BRD4 can be associated with the development of multiple cancers, and BRD4 is significantly associated with gliomas. The use of BRD4 inhibitors is a potential therapeutic target in gliomas and could be considered in such cases [26].

Our study diagnosed two cases as TRB, one of which was fit for molecular analysis and identified as PB-RB. Both cases, aged 4 years and 5 years respectively, developed disease progression during their induction chemotherapy, and none received RTH or stem cell transplant. Treating such a group of patients presents a challenge due to the risks of RTH and its effect on increasing the risk of subsequent malignancies in patients with germline RB mutations. Recent studies focus on possible therapies targeting RB mutated cancers [27]. Potentially sparing such groups the hazards of RTH by using targeted therapies will require further study to prove the efficacy and applications of such therapies.

Our study had a number of limitations. The scarcity of tissue materials obtained from biopsies led to exclusion of approximately half of the samples tested for methylation. This is mainly attributed to these tumor types where minimal biopsies are preferred to mitigate surgical risks. Consequently, the small number of patients included in each molecular subclass limited our ability for further statistical correlations and further analysis.

## Conclusion

Pineal parenchymal body tumors have diverse clinical and molecular backgrounds. Methylation analysis adds a different perspective to diagnosis and prognosis that can supplement traditional diagnostic methods, paving the road for new targeted therapies. Moreover, methylation analysis revealed new molecular entities in pineal parenchymal tumors.

## Supplementary Information

Supplementary Material 1.

## Data Availability

The datasets generated and/or analysed during the current study are available in the National Center for Biotechnology Information’s Gene Expression Omnibus (GEO: [http://www.ncbi.nlm.nih.gov/geo/] (http://www.ncbi.nlm.nih.gov/geo/)), accession number GSE269319).

## Abbreviations

WHO: World Health Organization
PTPR: Papillary Tumor of the Pineal Region
PPTID: Pineal Parenchymal Tumor of Intermediate Differentiation
PB: Pineoblastoma
DMT: Desmoplastic Myxoid Tumor
RTH: Radiotherapy
CSI: Craniospinal Irradiation
KBTBD4: Kelch repeat and BTB domain-containing protein 4
OS: Overall Survival
CCHE: Children’s Cancer Hospital Egypt
FFPE: Formalin-Fixed Paraffin-Embedded tissue
t-SNE: T-distributed Stochastic Neighbor Embedding
MNP: Molecular Neuropathology Classifier
GEO: Gene Expression Omnibus
MRI: Magnetic Resonance Imaging
GTR: Gross Total Resection
NTR: Near Total Resection
STR: Sub-Total Resection
CSF: Cerebro-Spinal Fluid
RB: Retinoblastoma
TRB: Trilateral Retinoblastoma
HDCT/AuHCR: High Dose Chemotherapy/Autologous Hematopoietic Cell Rescue
CR: Complete Response
PR: Partial Response
SD: Stable Disease
PD: Progressive Disease
EFS: Event Free Survival
IHC: Immunohistochemistry
BRD4: Bromodomain-containing protein 4
BET: Bromodomain and extra terminal
