# Supplementary material for: Descriptive and molecular analysis of pineal parenchymal tumors with clinical correlation
Source: BMC Cancer. 2025 Dec 6;26:100. doi: 10.1186/s12885-025-15331-1 (PMC12821833; doi:10.1186/s12885-025-15331-1)
Supplement: Supplementary file 1 — Supplementary Material 1. [file 12885_2025_15331_MOESM1_ESM.docx]

| **Patients’ Characteristics** | **Histopathological Diagnosis** | | | | | |
| --- | --- | --- | --- | --- | --- | --- |
|  | PB  (<3yrs) (n=9) | PB (≥3yrs) (n=30) | PTPR (n=5) | PPTID (n=2) | Pineocytoma (n=1) | TRB (n=2) |
| **Gender** |  |  |  |  |  |  |
| Males  Females | 6  3 | 19  21 | 3  2 | 2  - | -  1 | -  2 |
| **Metastatic status** |  |  |  |  |  |  |
| M+  M0 | 7  2 | 20  10 | -  5 | -  2 | -  1 | 2 |
| **Resection** |  |  |  |  |  |  |
| Biopsy  STR | 9  - | 27  3 | 5  - | 2  - | 1  - | 2  - |
| **Radiotherapy** |  |  |  |  |  |  |
| CSI Focal None  Unknown | 3 3 3 - | 27 - 1 2 | - 5 - - | - 1 1 - | - - 1 - | - - - - |
| **Disease Progression** |  |  |  |  |  |  |
| Local Local +Disseminated Disseminated | 1  5 | - 1 10 | 4  - | -  1 | -  - | - 1 - |
| **DNA-based methylation subclasses** |  |  |  |  |  |  |
| PB-miRNA1 | - | 10 | - | - | - | - |
| PB-RB1 | - | - | - | - | - | 1 |
| Retinoblastoma -MYCN | 1 | - | - | - | - | - |
| Papillary tumor of the pineal region | - | - | 1 | - | - | - |
| PPTID KBTBD4-altered | - | 1 | - | - | - | - |
| MB WNT | - | 1 | - | - | - | - |
| MB non WNT- non SHH | - | 1 | - | - | - | - |
| BRD4-LEUTX | - | 1 | - | - | - | - |
| N/A | - | 1 | 2 | - | - | - |

**Supplementary table 1:** Presenting the number of cases per each tumor group, clinical and molecular data within each group.

| DNA Methylation-Based Classification | ID | Prediction Score |
| --- | --- | --- |
| PB-miRNA altered 1 |  |  |
| PB, miRNA altered 1, Subclass A | Pin_02 | 0.69 |
|  | Pin_07 | 0.99 |
|  | Pin_10 | 0.94 |
|  | Pin_17 | 0.97 |
|  | Pin_22 | 0.97 |
|  | Pin_24 | 0.94 |
|  | Pin_32 | 0.96 |
|  | Pin_34 | 0.99 |
| PB, miRNA altered 1, Subclass B | Pin_15 | 0.99 |
|  | Pin_31 | 0.98 |
| PB- RB1 | Pin_08 | 0.99 |
| RB, MYCN Activated | Pin_09 | 0.95 |
| PTPR, Subtype B | Pin_33 | 0.98 |
| PPTID, KBTBD4 Altered, Subtype A | Pin_21 | 0.83 |
| MB, WNT Activated | Pin_20 | 0.99 |
| MB- non WNT non SHH, Group 3, Subclass II | Pin_14 | 0.63 |
| CNS embryonal tumor with  BRD4: LEUTX fusion | Pin_05 | 0.99 |
| N/A | Pin_04 | No match |
|  | Pin_11 | No match |
|  | Pin_12 | No match |

**Supplementary table 2**: Generated prediction scores for each DNA methylation-based subclass, by Heidelberg classifier 12.8.


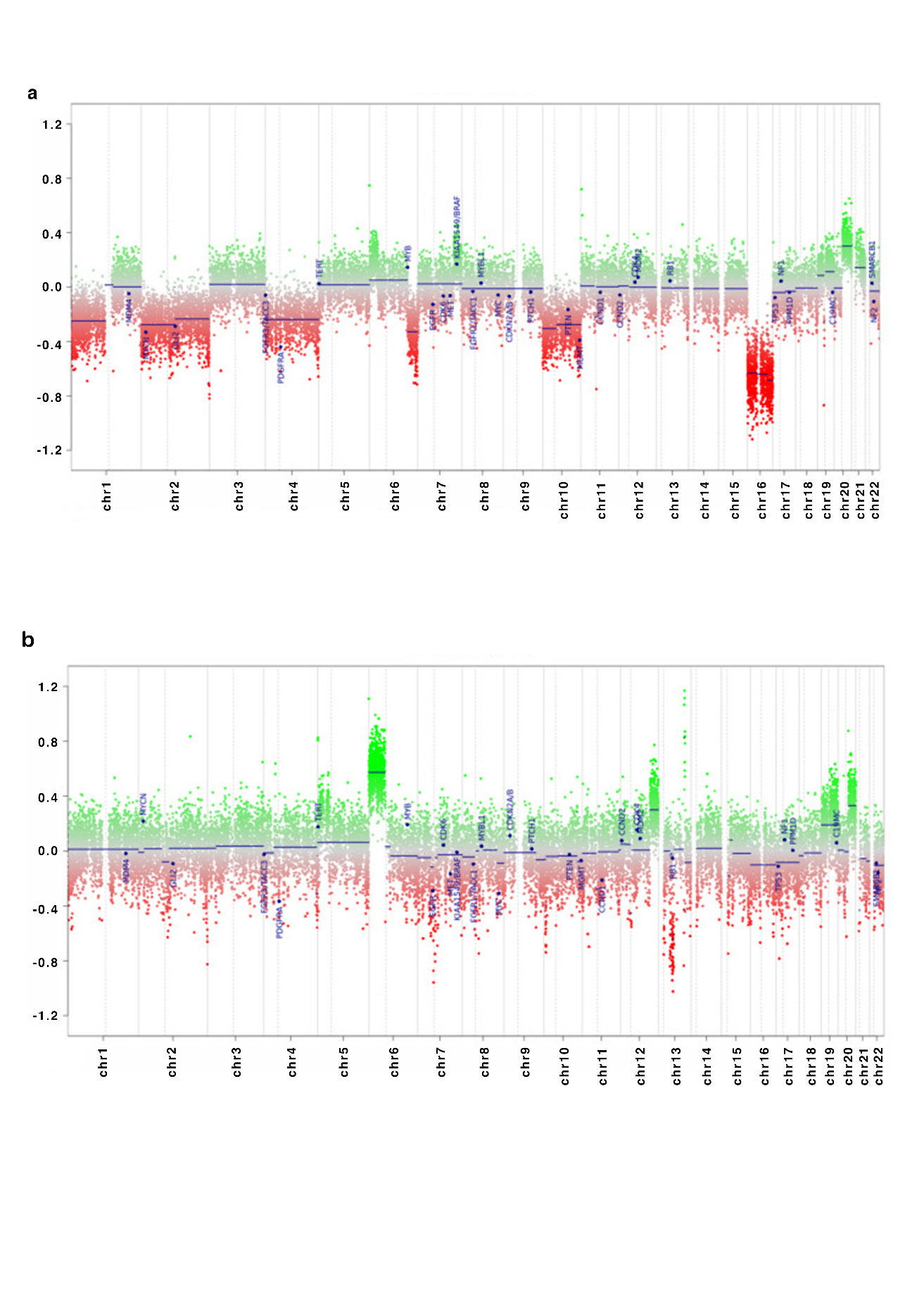


**Supplementary figure 1:** Copy Number Variation (CNV) profiles. (**a)** PB-RB1 showing loss of ch16. (**b)** Retinoblastoma-MYCN displayed ch6p gain.

**
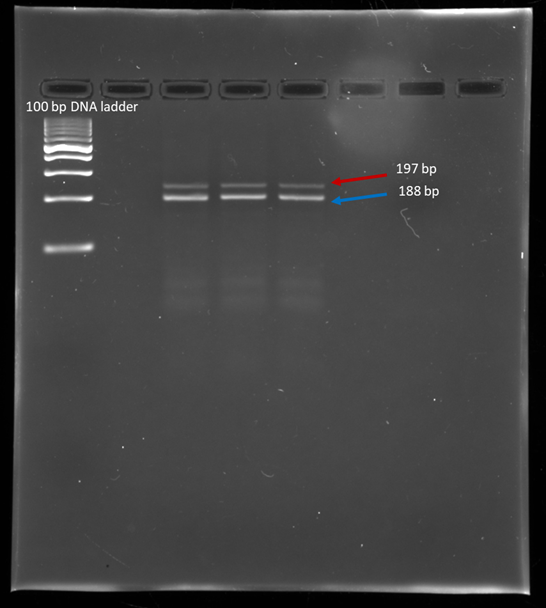
**

**Supplementary figure 2: Agarose gel electrophoresis showing PCR amplified from genomic DNA of sample pin_21 to detect KBTBD4 insertion.**Using a 100 bp DNA ladder for size estimation, it was shown that the wild type allele produces a band of 188 bp (blue arrow), while the mutant allele with the insertion results in a larger band of 197 bp (red arrow).
